# Exploring the provision of hospital trauma care for road traffic injury victims in Iran: a qualitative approach

**DOI:** 10.5249/jivr.v5i1.195

**Published:** 2013-01

**Authors:** Hassan Haghparast-Bidgoli, Hamidreza Khankeh, Eva Johansson, Mohammad Hossein Yarmohammadian, Marie Hasselberg

**Affiliations:** ^*a*^Health Management and Economics Research Centre, Faculty of Management and Informatics, Isfahan University of Medical Sciences, Isfahan, Iran.; ^*b*^Division of Global Health, Department of Public Health Sciences, Karolinska Institute, Stockholm, Sweden.; ^*c*^The Swedish Research School for Global Health, Partnership between Umeå University and Karolinska Institute, Sweden.; ^*d*^Department of Nursing, University of Social Welfare and Rehabilitation, Tehran, Iran.; ^*e*^Department of Clinical Science and Education, Karolinska Institute, Stockholm, Sweden.; ^*f*^Nordic School of Public Health, Gothenburg, Sweden.

**Keywords:** Trauma care, Emergency department, Road traffic injuries, Paradigm model, Iran

## Abstract

**Background:**

Identifying factors affecting the provision of trauma care is essential for improving the quality of care for road traffic injury (RTI) victims. The study aimed to explore the perceptions and experiences of trauma care among injured patients and health professionals to identify factors influencing an effective trauma care delivery at emergency departments (EDs) in Iran.

**Methods:**

The study was conducted with a grounded theory approach. The study participants consisted of 15 health professionals and 20 injured patients. The data were collected via semi-structured interviews and were analyzed using constant comparative analysis method.

**Results:**

Lack of a systematic approach to providing trauma care at EDs emerged as the core category. The leading factors in the development of the core category were unclear national policies and poor organization of care at the ED. Other major factors were contextual factors in the environment of the hospitals such as inappropriate structure and unsupportive environment and also factors specific to the context of Iran such as a rapid increase in the number of traumas. Professionals reacted to the prevailing conditions in ways that contributed to an ineffective trauma care, even though strategies employed by Emergency Medicine Physicians (EMPs) improved the quality of trauma care locally.

**Conclusions:**

Building a national trauma system, using available professional resources especially EMPs, and implementing low cost and evidence-based improvements such as establishing trauma teams and trauma training for staff working at the EDs on a regular basis is necessary in order to improve delivery of trauma care at the hospitals.

## Introduction

Injuries are one of the leading causes of mortality in the world, with more than five million deaths each year and even more disabilities.^1,2^ Road traffic injuries (RTIs) constitute the majority of deaths caused by injuries.^3^ In low and middle-income countries (LMIC) RTIs account for as much as 90% of Disability Adjusted Life Years (DALYs) lost and for 90% of deaths.^3,[Bibr B4]4^ These countries in general are experiencing a rapid urbanisation and motorisation, resulting in an increased exposure to risk factors for RTIs, such as unsafe public transportation, high speed, and a diverse vehicle mix on the roads.^1^

In addition, studies have shown that an inadequate public health infrastructure, including trauma care, in LMICs has an important role in death and disability from RTIs.^1,5^ Evidence from both high income countries (HICs) and LMICs suggests that a significant proportion of the deaths and disabilities caused by RTIs could be eliminated by improvements in the organisation of trauma care.^2,6-9^

Iran has one of the highest RTI death rates among LMICs.^10-12^ RTIs are considered to be the second highest cause of mortality in Iran after cardiovascular diseases.^10^ Studies in Iran have shown that about 60% of the deaths occurred at the crash scene or on the way to hospital and more than 30 % at hospital.^11,13,14^

Previous studies on trauma care in Iran have mainly focused on pre-hospital trauma care,^15-20^ while studies investigating care provided for injuries at hospitals are rare. The studies done so far about hospital trauma care in Iran are mainly epidemiological ones with a focus on demographical characteristics of patients and mechanisms of traumas.^14,21,22^ Moreover, the few studies that have been conducted on trauma care at hospitals in other LMICs have mainly concentrated on the availability of resources and interventions for improving outcome and quality of trauma care at hospitals.^2,6,23,24^

This explorative study was designed with the aim of identifying factors influencing the provision of trauma care at emergency departments (EDs) from the perspective of injured patients and health professionals. We have used a qualitative approach in the study to get in-depth information about the trauma care process at EDs and factors influencing this process.

## Methods

**Design**

Data were collected using semi-structured interviews and analyzed based on a grounded theory approach as described by Strauss and Corbin, using the Paradigm Model.^25^ One of the main aims of grounded theory is to generate hypotheses, theories, and tentative models based on empirical data.^25^ This approach is considered to be suitable when a researcher investigates a new area or intends to explore a known area from a fresh perspective.^25,26^

The research team consisted of a triangulation of researchers with different backgrounds from Iran and Sweden; one sociologist (M.H) with experience of injury research, two nurses (E.J and H.K) with expertise in emergency care, public health and qualitative research, one health economist (principal investigator, H.H.B) active in studies of road traffic injuries and trauma care and one experienced researcher in emergency care and disaster management (MHY).

**Study setting**

The study was conducted in Tehran, the capital city of Iran. It is the most populated city in the country with about 13 million inhabitants.^27^ Based on unofficial data, it is estimated that there are more than five million automobiles and two million motorcycles in Tehran. Moreover, every day more than 1000 new automobiles (annually about 300,000) and more than 500 new motorcycles are registered and added to the number of vehicles on the road.^27^ Despite the fact that several new dual carriageways have been constructed in the city during recent years, the increased number of vehicles has exceeded the additional capacity provided by these.^22^ Overcrowded roads are one of the reasons for the high number of accidents in the city.^22^ In 2010, nearly 40,000 people were injured and more than 2300 died due to road traffic crashes in Tehran.^28^

There are more than 100 small and large hospitals that provide different levels of trauma care in Tehran.^29^ Currently there is no established trauma care system in the city and no hospital has been designated as a Level I trauma centre. However, a few teaching hospitals have tertiary medical care facilities.^29^

**Study participants and data collection **

The study participants consisted of 15 health professionals and 20 injured patients. The inclusion criteria for selecting the medical professionals was that they should have at least three years experience in providing trauma care (pre-hospital or hospital trauma care). The criteria for injured patients was that they should be all male motorcycle drivers injured in a traffic crash and admitted to the orthopedic departments at the selected hospitals. The reason for including only motorcyclists in the study was that the majority of RTI victims admitted to hospitals in Iran are motorcyclists.^10^ Since men in Iran dominate motorcycle driving, all the injured motorcyclists were male. The health professionals included seven professionals from the Emergency Medical Services (EMS) and eight professionals from the EDs of five hospitals providing trauma care (Table 1). The injured patients included 20 patients (17 to 57 years old) with motorcycle injuries and treated in the orthopaedic departments at three different hospitals. Selection of the professionals and injured patients initially started using a purposive sampling technique and according to the emerging codes and categories, continued using a theoretical sampling technique to clarify and develop explored concepts and to saturate them. 

**Table:** Table 1: **Medical professional characteristics**

	No.	Years of experience in trauma care(min-max)	Male	Female
EMP*	2	8-13	2	0
Physician	2	4-10	2	0
Nurse	9	6-15	7	2
Surgeon	2	10-15	2	0

* Emergency Medicine Physician

The data were collected via semi-structured interviews at the five hospitals with the greatest load of trauma patients in Tehran. All interviews were conducted in Persian. The interviews with the professionals began with general questions about their own experience of providing trauma care for RTI victims and their perceptions of “factors influencing (inhibiting or facilitating) an effective provision of trauma care in the ED of the hospitals”. Probing questions were also used to clarify information and to gain additional data. The interviews with the professionals lasted from 20 to 100 minutes. The interviews with the injured patients were mainly focused on the process of care. 

Additionally, four observations were done at the EDs in three hospitals as a validation of the findings of the study. Field notes were taken during the observations. All interviews and observations were done between January and April 2009 by the first author (H.H.B.).

Ethical approval of the study was obtained from the National Ethics Committee of the Ministry of Health in Iran. Verbal consent was given and all participants were informed of the purpose of the study and that they could refuse to participate or withdraw from the interviews at any time.

**Data analysis**

All interviews were recorded, transcribed verbatim and analyzed based on a grounded theory approach, using the Paradigm model.^25^ Accordingly, data collection and data analysis took place simultaneously. We conducted an initial analysis of each interview before the next interview and if some important issues emerged, these were then brought up in the next interview. 

Following Strauss and Corbin’s recommendations, data analysis was performed at three levels including open, axial and selective coding.^25^ Open coding consisted of a line by line scrutiny of the data in order to discover the codes expressed by the participants and then labelling and grouping of similar codes into categories and sub-categories. The focus of axial coding was on further conceptualization of the categories by specifying the relationships between them. At the selective coding stage one core category which related to all other categories was identified. We used the Paradigm Model in order to explore the relationships between concepts.^25^ The basic purpose of the Paradigm model is to enable the researcher to think systematically about data and relate categories and concepts in complex ways.^30^ The basic components of the Paradigm model technique include: causal conditions, contextual conditions, actions and interactions or strategies taken in response to the phenomena and intervening conditions that assist or hinder interactions and the consequences of the actions.^25,30^

The analyses were done by the first author (H.H.B.) in collaboration with the co-authors.

**Rigour**

Credibility was ensured through several procedures. First, constant comparison was done in order to verify the emergent codes and categories and to develop new categories.^26^Second, a summary of the primary results was discussed with some of the participants to check whether the results were in accordance with their experiences (member check). Third, emergent codes and categories were discussed continuously between the main author and the co-authors, some with extensive expertise in qualitative methods (Peer check or debriefing).^31^ Finally, triangulation of researchers helped to take into account different perspectives when analyzing the data.

**Findings**

**Figure 1:Organization of data (concepts and categories), using the Paradigm Model:**
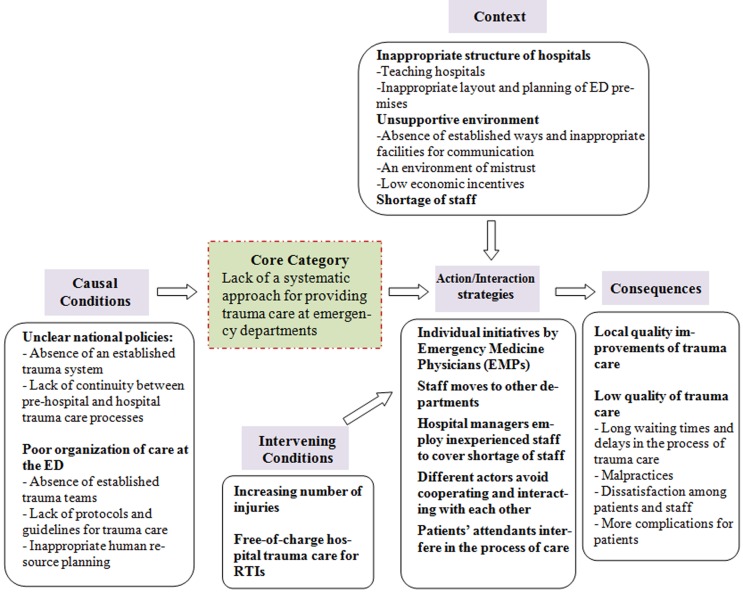


In this study “Lack of a systematic approach for providing trauma care at emergency departments” was identified as the core category. It reflects the views of the participants regarding factors inhibiting the provision of effective trauma care at hospitals. This was seen at different levels, from the national level to the hospital level. Figure 1 illustrates how the core category is related to the concepts and categories identified in the study. The findings will be described according to Figure 1.

**Causal conditions**

In this study, the causal conditions are seen as events or happenings that lead to the occurrence or development of a non-systematic trauma care delivery. Unclear national policies and poor organization of care at the EDs were perceived as main issues hindering a systematic approach for providing trauma care. 

There is no existing national trauma care system in the country, which could develop rules and regulations for organizing trauma care and coordinate all activities within the area. It was also reported that the pre-hospital and hospital trauma care processes are not integrated with each other, which was seen as hindering the continuity of care from the crash scene to the ED. One of the reasons for this, mentioned by the participants, was the separated and independent management systems of the pre-hospital and hospital trauma care.

“Pre-hospital and hospital processes are separated from each other. When we transfer a patient to the ED, they start to examine the patient again to decide what treatment the patient needs.... The process of treatment should start in the ambulance and continue in the hospital...”. (Professional 5)

Trauma teams have not been established in the EDs of hospitals providing trauma care. However, some hospitals consider having a Cardio Pulmonary Resuscitation (CPR) team to be a substitute for a trauma team. According to the participants, most hospitals providing trauma care have established triage units. However, these units are mostly active in hospitals where an EMP team is in charge of the ED. The participants also emphasized the lack of protocols and guidelines for many diseases especially for traumas.

“We don’t have trauma teams in the hospitals, even if hospitals have a CPR team, which include people from different wards, these teams are not located in the ED and they are not always available”. (Professional 5) 

Inappropriate human resource planning was perceived as another factor contributing to poor organization of trauma care delivery. The participants implied that the allocation of ED staff, especially nurses, is not based on patient load, but instead based on a fixed number of staff. The participants also mentioned, that staff are not selected based on skills and experiences. They discussed that students and newly graduated staff without practical experience of emergency care are employed to work in the ED. This is often done with the purpose of giving the staff experience and often they are replaced after a short period of time.

 “Unfortunately, despite the regulations by the Ministry of Health, which require that the most skilful staff work in the ED, newly graduated students or staff without any experience are sent to the ED to get experience of different types of conditions ... and after three to six months when they have enough experience, they are moved to other wards”. (Professional 8)

“One of the interns who was examining me couldn’t take a blood sample and my aunt, who is a nurse, came and did the test instead”. (Patient 3)

Lack of or inefficient trauma-related training courses were perceived as another important barrier, which resulted in inadequate knowledge and skills of staff working in the ED. However, the participants noted that some trauma-related courses, such as Advanced Trauma Life Support (ATLS) have been established by a few hospitals, especially where an EMP team is in charge. These courses are mainly given for physicians and are not offered on a regular basis.

“The training courses are given periodically every six months or once a year. They don’t take into consideration the number of new staff and who needs training…”. (Professional 8)

**Context (contextual conditions)**

Contextual conditions are a set of conditions that create circumstances or problems through which groups or individuals respond by their actions/interactions.^25^ The participants described some contextual factors, which affected a systematic process of trauma care delivery and also influenced the strategies chosen.

The participants reported that almost all of the hospitals providing trauma care were teaching hospitals. This meant that various professional groups visited the patients at different points in time. Repetition of activities and conflicts between educational and non-educational groups were mentioned as common problems in the teaching hospitals. 

“In a teaching hospital different educational groups are involved in treating patients.... Each patient is treated by several medical experts and each of them needs some time with the patient”. (Professional 8) 

“In the ED, several medical students came at different points in time to examine me and asked some questions and then left without coming back....”.(Patient 3)

The participants also emphasized that access to other departments (such as MRI, CT-scan, laboratories, ICU and surgery), which are highly utilized by the ED, could be difficult for example, because of long distances. Lack of infrastructure for helicopter landing, shortage of physical space and ineffective design of the ED were other important barriers discussed by the participants. The sub-standard Trauma and CPR rooms with inappropriate physical access are some examples of ineffective design of the ED.

“As the hospital building is very old, the CT-scan is far from the ED located on the other side of the hospital…. This has a significant effect on the care provided for patients and causes dissatisfaction”. (Professional 14)

“The CT-scan is far from the ED and I had to go there two times. Every time unskilled staff moved me to a usual stretcher and transferred me… I had several fractures and also internal problem and a lot of pain…”. (Patient 19)

The participants described the ED as an unsupportive environment. One example was the absence of established ways for communication and interaction between different actors involved in trauma care at the ED. This was also the case between different hospitals, especially for transferring patients. Insufficient trust among different actors involved in providing trauma care was another important factor contributing to the unsupportive environment. The participants commented that there is a lack of trust between different specialist groups in the ED and between the ED and EMS staff. Mistrust between the EDs and EMS staff was partly perceived as lack of knowledge about each other’s duties, qualifications and skills. 

“ED staff usually don’t ask for and don’t trust our medical reports and activities done for the patients. It depends on the hospital. Some hospitals, where EMP teams are in charge of the EDs ask for our reports and the procedures we have done for the patients”. (Professional 2)

The participants also discussed that patients and their attendants (including family and friends) often do not trust the ED staff. They argued that this is mainly because they lack knowledge about the EDs work.

“I asked the ambulance staff to bring me to this hospital because I don’t trust the staff of hospital X. My family had a bad experience when they went there last year”. (Patient 1)

The participants described the EDs as a stressful environment with a high workload, mainly due to the high load of critically ill patients and problems in dealing directly with patients’ attendants. These issues along with low economic incentives were seen as the main concerns of the staff.

“A lot of staff don’t like to work in the ED because it is a stressful environment, always busy and the salary is lower compared to other wards in the hospital”. (Professional 12)

The shortage of professional and non-professional staff was another important contextual problem that was emphasized by most participants. The participants argued that restrictions regarding the employment of professional staff are one common problem that all hospitals face. 

“Shortage of professional staff especially nurses and non-professional staff such as nurse assistants and staff for transferring patients, is clearly apparent”.(Professional 10)

“My family transferred me to the laboratory and radiology wards when I was in the ED because they said they didn’t have staff to transfer me”. (Patient 6)

**Intervening conditions**

In this study, the intervening conditions are the broader structural context affecting the trauma care delivery system. These conditions either facilitate or constrain the action/interactional strategies taken within a specific context.^25^ Factors, such as an increasing number of injuries and providing free-of-charge hospital trauma care for RTI victims could be seen as intervening conditions.

According to the participants, the number of injures have increased dramatically during recent years and this has affected the patient load of the hospitals and also the demand on trauma care. The participants also mentioned that one reason for the increasing demand for hospital trauma care is raising the number of patients transported by EMS which is a result of the substantial improvements in the pre-hospital trauma system in the country.

“The number of traumas has increased during recent years, and moreover, because of improvements in the EMS system, they transfer more patients to the hospitals than before, but the number of public hospitals has not increased for 30 years”. (Professional 9)

Based on a newly approved national policy, all medical services provided in the hospitals for RTI victims are now funded by the government. This policy, according to the participants, has improved accessibility to and utilization of hospital trauma care for RTI victims. The participants also mentioned that this policy has improved the financial stability of hospitals and resulted in improved delivery of trauma care at the hospitals.

“All medical services are free-of-charge for RTI victims. This policy has helped hospitals in terms of financial support and improving care”. (Professional 14)

**Actions/ Interaction strategies**

Action/interaction strategies are purposeful or deliberate course of actions, which are taken by individuals or groups in response to events, problems or issues which occur under certain conditions.^25^ The participants described that different actors involved in providing trauma care react in different ways in order to deal with factors influencing the trauma care delivery. 

Some hospitals have appointed an EMP team to be in charge of the ED. EMP teams have made changes in the local organization of trauma care, developed some training courses for staff and also improved facilities and equipments of the ED.

“The EMP team provides all medical procedures a patient needs and depending on the condition of the patient, consultations from other specialist groups in the hospitals are requested. This shortens the process of care and saves a lot of time”. (Professional 8)

“Since the adding of emergency medicine specialists to the ED, trauma care has improved in terms of appropriate equipment and trained staff and training program”. (Professional 10)

The participants argued that a lot of staff change their job or move to other wards, mainly because of factors such as a high work load, a stressful environment and low economic incentives.

“A lot of staff change their job after a short period of time, because the ED is always busy and is a very stressful environment especially because of patients’ attendants”. (Professional 11)

In response to the shortage of staff in the EDs, the hospital managers generally employ newly graduated staff or staff without any experience and training in emergency care. In some cases this was done in order to compensate for the shortage of staff by means of short-term contracts with professional staff, especially nurses.

“We usually cover shortage of staff by employing newly graduated nurses and short-term contract staff”. (Professional 12)

In reaction to the absence of established ways and also facilities for interaction and communication some actors try to avoid interacting and cooperating with others. This was seen between professionals working in the EDs and also between the EDs and other departments such as laboratories, and X-ray.

“Because there is not a coordinated and united system in the ED, different specialist groups and wards do not cooperate with each other for providing care...”. (Professional 8)

In response to the limitations of the communication system between EMS and the ED, EMS do not notify the ED before transferring patients to the hospital (except for some critical cases) and this often causes conflict between the ED and EMS staff. It was also discussed that EMS staff usually transfer patients to the hospitals with which they have good relationship. This was especially the case when hospitals do not trust EMS staff or avoid admitting trauma patients which are referred by the EMS. The participants argued that this was true especially for multiple trauma patients because of their complications and their long hospital stays.

“…. Sometimes EMS ambulances send several critical patients at the same time or one after the other without informing the ED …”. (Professional 13) 

“We usually don’t transfer patients to those hospitals where the ED staff do not trust our staff and will try to avoid admitting the patients transported by our staff. We usually transfer patients to the hospitals that have a good relationship with the EMS.” (Professional 1)

Moreover, the participants noted that the patients’ attendants interfere with the process of trauma care, mainly with the intention of helping the patients. They argued that interference by the patients’ attendants is mainly due to reasons such as lack of knowledge of how to communicate and interact with the ED staff, cultural values (willingness to help), insufficient trust in the ED staff and shortage of staff. The participants also discussed that there is no plan for improving communication and interaction between the ED staff and patients’ attendants.

“When I was in the ED my family transferred me to the laboratory and to the radiology wards because they said they don’t have staff to transfer me”. (Patient 6)

**Consequences **

The consequences are outcomes of the action / interaction strategies chosen by the actors.^25^ The strategies employed by different actors involved in providing trauma care at the EDs have, in some cases, improved the quality of trauma care. For example, in the hospitals where an EMP team is in charge of the ED, the local changes which they have made in order to deal with the situation have improved the quality of trauma care. This has resulted in shorter waiting and treatment time, and increased satisfaction among patients. 

On the other hand, strategies such as changing job (high turnover of personnel), employing inexperienced staff, interfering from patients’ attendants and avoiding interaction and cooperation have had negative effects on the quality of trauma care in most hospitals. For example, high turn-over of staff and employment of inexperienced and unqualified staff in order to compensate for the shortage and also high turn-over of staff generally resulted in delays and prolonged treatments, and caused dissatisfaction among patients, their attendants and staff. These factors have also, in some cases, caused malpractices and increased adverse effects among patients. 

Interference of patients’ attendants, which was largely the consequences of staff shortages and the employment of unqualified staff, mainly interrupted the process of care. This interference also caused distress and dissatisfaction among staff. Moreover, avoiding interaction and cooperation between the actors, which was mainly seen between different professional groups in the ED and between the ED and EMS staff, resulted in long waiting times and delays in the process of delivery of trauma care and caused dissatisfaction among the patients. 

## Discussion

The findings of this study indicate that the main barrier to providing effective trauma care is the lack of a systematic approach. Unclear national policies and poor organization of trauma care delivery at the hospitals were identified as the main factors contributing to a non-systematic approach to trauma care provision. Other influential factors were related to the context of the hospitals such as inappropriate structure and unsupportive environment and also country-specific factors for Iran such as a rapid increase in the number of traumas. Various strategies employed in order to deal with the conditions resulted in ineffective trauma care, although strategies employed by EMPs improved the quality of trauma care locally. The discussion will mainly focus on causal and contextual conditions.

**Absence of a national trauma system**

Absence of an organized national trauma system was one of the main issues highlighted by the participants in the current study. This issue has also been mentioned in a few previous studies in Iran.^29,32,33^ Evidence, mostly from high-income countries, indicates that a well-organized trauma care system can reduce trauma deaths and disabilities substantially.^2,6,7^ The development of such a system is important, especially in a country such as Iran, where injuries, particularly RTIs, are one of the leading causes of mortality and morbidity.^34^

**Continuity of the trauma care process**

Reducing injury-related mortality and disability, especially RTIs, requires an integrated system of care with effective initial assessment and treatment at the scene of the incident, followed by efficient transport to hospital, and high quality care in EDs, in-hospital care and rehabilitation.^7,35^ It should be considered that all stages of care require integration. This is especially important in Iran, since pre-hospital (or EMS) and hospital trauma systems are separated from each other and this interrupted continuity of trauma care from pre-hospital care to hospital care. 

**Poor organization of trauma care at EDs**

Poor organization of trauma care at EDs was identified as one of the main barriers to providing effective trauma care, which was partly affected by lack of an organized national trauma system. Some of the issues that contributed to poor organization of trauma care at EDs included absence of trauma teams, lack of protocols and guidelines for trauma care and inappropriate human resource planning (including recruiting and training). Most studies have confirmed substantial reduction in mortality as a result of improvements in the hospital’s organization of trauma care.^2,6,8,9^ Two main areas which require improvement are the establishment of trauma teams and training of staff working in the EDs. 

In Iran, there are no organized trauma teams in the hospitals providing trauma care, although, in some hospitals a team of specialists, who often are in charge of the ED, such as general surgeons are designated to lead delivery of trauma care at the hospital. Organized trauma teams have been shown to improve the process and outcome of trauma care, in both high-income and LMICs.^2,6^ For example, establishment of a trauma team at an urban trauma centre in Turkey showed that, in addition to reducing unexpected deaths, deaths due to severe injuries were decreased by 10%. These improvements were perceived as being caused particularly by improved resuscitation and airway management.^24^

Another important area that needs to be strengthened is training of professional staff, especially doctors and nurses. This should include both formal (during basic and postgraduate) and continuing education.^6,9^ A modified ATLS course has been developed in recent years in Iran and has been implemented for professional staff in some hospitals, especially those hospitals where EMPs are in charge of trauma care. However, this course was mainly implemented for physicians and it is not part of a routine training course for ED staff. Continuing education courses have been shown to improve the process and outcome of trauma care at hospitals.^2,6,9^ For example, Ali et al.^23,36^ reported that a two-day continuing education course (ATLS) in Trinidad reduced the mortality of severely injured patients attending hospital, from 68% before the training period to 34% after the training.

**Limitations in structure of the hospitals and unsupportive environment**

There are several contextual factors, which indirectly and in combination with the factors discussed above influence an effective provision of trauma care. Two of the main factors are limitations in the structure of hospitals and unsupportive environments, which influence the whole process of trauma care delivery. The hospitals that provide trauma care are mainly teaching hospitals where different educational and non-educational groups are involved in providing trauma care and problems such as long waiting and treatment times, repetition of activities and conflicts between different groups are common. Inappropriate layout and design of ED premises in the hospitals is another barrier, which limited accessibility to other departments. This issue has previously been mentioned in a local study in Iran.^37^ To improve the infrastructure might be difficult in the short run since most hospitals are old and to improve their physical structure would be very expensive. 

Unsupportive environments were mainly reflected in the study by the absence of plans or arrangements for improving communication and interaction between different actors (for example between EMS and the EDs or between different professional groups at the EDs), mistrust among different actors and low economic incentives for staff working at the EDs. These issues could, to some extent, be improved by establishing a well-organized, protocol-regulated trauma system and by clarifying the role of different actors. It also requires a better communication and interaction between different actors through defined and established ways for communication. 

**Uneven development of the services at EMS and at the hospitals**

Another important issue in the context of Iran is the uneven development of the services at EMS and at the hospitals. While EMS has improved significantly (in terms of physical and human resources) during recent years,^15,16,38^ the number of hospitals providing trauma care has not changed for a long period of time. In addition, hospitals have not been classified for providing trauma care (trauma centre levels) and a standard trauma centre has not been established yet.^33^ This has contributed to an increased number of patients transferred by EMS to a few tertiary teaching hospitals which are delegated to provide trauma care, even though they already have a high load of medical patients. The new legislation on free-of-charge medical care for RTI victims has improved the access to trauma care for all people, but has also increased the load of injured patients referred to the hospitals. 

**Role of Emergency Medicine Physicians (EMPs)**

One of the advantages of the health care system in Iran is the existence of Emergency Medicine as a specialty. Few LMIC have specialists in emergency medicine, and in many countries this is not recognized as a specialty of its own.^7^ The specialty was developed in Iran in 2000^39^ and graduated EMPs have been involved in providing trauma care for several years. The findings of our study showed that the EMPs have improved the delivery of trauma care in their own ED. The positive influences of EMPs on the quality of pre-hospital trauma care have also been reported in a previous study.^16^

**Strengths and limitations**

The current study, which is one of few qualitative studies in this area, explored different aspects of trauma care delivery at EDs from the perspective of both patients and professionals. Employing the paradigm model in the study helped to explore and better understand complex relationships between identified concepts and categories. In order to increase credibility and the consistency of the findings, different methods including the constant comparison method, member check, and peer review were employed. The study was conducted in an Iranian context; however, its findings may be applied to other LMICs with a similar context.

## Conclusion

Lack of a systematic approach for providing trauma care was identified as the main barrier to effective provision of trauma care at the EDs. Effective provision of trauma care at the EDs requires improvements in a number of crucial areas at the national and hospital levels. Building a national trauma system, using available professional resources especially EMPs, is necessary in order to regulate provision of trauma care at all levels of care (from pre-hospital to rehabilitation) and between different actors. Implementing low cost and evidence-based improvements such as establishing trauma teams and trauma training courses (including ATLS) for staff working at the EDs on a regular basis is needed in order to improve delivery of trauma care at the hospitals. Further more, it is necessary to provide a supportive environment for staff working in the EDs and other actors involved in the delivery of trauma care at the EDs, especially through improved communication and interaction between them.
